# Clinical heterogeneity of Pisa syndrome across Lewy body disorders: a multicenter comparison of Parkinson’s disease and dementia with Lewy bodies

**DOI:** 10.3389/fnagi.2026.1856771

**Published:** 2026-07-15

**Authors:** Zhou Su, Mengran Liu, Jun Kuai, Xinran Bao, Xia Guo, Meimei Zuo, Hao Wu, Jinghuan Gan, Shuai Liu, Yong Ji

**Affiliations:** 1 Department of Neurology, The First Affiliated Hospital of Henan Medical University, Xinxiang, Henan, China; 2 Henan Medical Key Laboratory of Neurology, Henan Joint International Laboratory of Neurorestoratology for Senile Dementia, Henan Key Laboratory of Neurorestoratology and Protein Modification, Xinxiang, Henan, China; 3 School of Education, University of Bristol, Bristol, United Kingdom; 4 Department of Gastroenterology, The First Affiliated Hospital of Henan Medical University, Xinxiang, Henan, China; 5 Department of Neurology, First Hospital of Qinhuangdao, Hebei, China; 6 Department of Neurology, The First Affiliated Hospital of Baotou Medical College, Baotou, China; 7 Department of Neurology, Cangzhou People’s Hospital, Cangzhou, China; 8 Tianjin Key Laboratory of Cerebrovascular and of Neurodegenerative Diseases, Department of Neurology, Tianjin Huanhu Hospital, Tianjin Dementia Institute, Tianjin, China; 9 Department of Neurology, Beijing Friendship Hospital, Capital Medical University, Beijing, China

**Keywords:** dementia with Lewy bodies, Parkinson’s disease, Pisa syndrome, gait, neuropsychiatric symptoms

## Abstract

**Background:**

Pisa syndrome (PS) is well recognized in Parkinson’s disease (PD) but has been less studied in dementia with Lewy bodies (DLB), and direct comparisons are scarce. The aim of this study is to compare the clinical expression of PS across PD and DLB.

**Methods:**

In this multicenter cross-sectional study, patients were classified as PD without PS (*n* = 50), PD with PS (*n* = 41), DLB without PS (*n* = 45), or DLB with PS (*n* = 35). Standardized assessments covered cognition, neuropsychiatric symptoms, caregiver burden, motor severity, gait, falls, daily function. Two-way models, disease-specific logistic regression, and mediation analyses were performed.

**Results:**

In both disorders, PS was associated with longer disease duration, worse daily functioning, and more recurrent falls. Significant disease × PS interactions were observed across cognitive, neuropsychiatric, gait, and fall outcomes. DLB-PS carried the heaviest cognitive and neuropsychiatric burden, whereas PD-PS showed the greatest motor and gait impairment. In PD, PS was independently associated with disease duration, MDS-UPDRS-III score, gait symmetry, and stride length. In DLB, PS was independently associated with lower attention and visuospatial/executive scores, greater hallucination severity, and a higher CDR stage. Attention/visuospatial dysfunction partly mediated the association between PS and recurrent falls in DLB, whereas gait asymmetry/stride impairment partly mediated the same association in PD.

**Conclusion:**

PS is not a uniform phenotype across Lewy body disorders. Our findings suggest that it behaves relatively more like a cognition-neuropsychiatry-associated axial syndrome in DLB, whereas in PD, it shows stronger associations with motor and gait features. The effect sizes are modest, indicating that PS is a multifactorial clinical sign rather than a strong predictor of any single domain.

## Introduction

Pisa syndrome (PS) is an axial postural deformity characterized by marked lateral flexion of the trunk that typically worsens in the upright position and improves, at least partially, when the patient lies supine or undergoes passive mobilization (Doherty et al., 2013; Barone et al., 2016). In Parkinson’s disease (PD), PS appears to reflect a convergence of asymmetric basal ganglia dysfunction, altered sensory integration, disturbed perception of body verticality, musculoskeletal adaptation, and medication-related factors (Castrioto et al., 2014; Barone et al., 2016; Tinazzi et al., 2016).

Dementia with Lewy bodies (DLB) and PD belong to the category of Lewy body disorders, which share alpha-synuclein pathology and overlap substantially in their cognitive, motor, neuropsychiatric, sleep, and autonomic manifestations (McKeith et al., 2017; Taylor et al., 2020). That overlap makes DLB an especially informative setting in which to ask whether PS represents the same syndrome seen in PD or whether it acquires a different clinical meaning once cognitive and neuropsychiatric dysfunction become central to the disease phenotype.

In DLB patients, PS was associated with longer disease duration, a higher frequency of parkinsonism, a more advanced dementia stage, greater impairment in activities of daily living, and selective cognitive deficits (Su et al., 2022), and a heavier neuropsychiatric and caregiver burden (Su et al., 2025a). These observations argue against a narrow interpretation of PS in DLB as a postural complication. Instrumented gait analysis in DLB demonstrated that slower gait speed, shorter stride length, and reduced gait symmetry track with lower cognitive performance and independently predict fall risk (Su et al., 2025c). Resting-state functional MRI suggested that DLB-related cognitive impairment is underpinned by disrupted default mode and visual network connectivity, reduced posterior occipital and parietal activity, and impaired global network efficiency (Su et al., 2025b). These studies support the idea that PS may sit within the cognition-neuropsychiatry-gait syndrome rather than within a purely motor axis in DLB.

Most studies in PD and DLB with PS have been conducted separately. However, the field lacks a harmonized comparison capable of disentangling what is shared and what is disease-specific. The present multicenter study was therefore designed to compare the clinical phenotype of PS across PD and DLB using a common assessment framework. The primary objective was to examine disease-by-PS interaction effects on attention, visuospatial/executive performance, neuropsychiatric symptoms, gait measures, and fall burden. Secondary objectives were to identify predictors of PS within PD and DLB separately and to explore whether cognitive or neuropsychiatric measures mediate the relationship between PS and clinically meaningful outcomes. We hypothesized that PS would be associated with poorer attention and visuospatial function and worse daily functioning in both diseases but that DLB-PS would show a relatively stronger neuropsychiatric and caregiver-burden signature, whereas PD-PS would show a relatively stronger motor-sensorimotor signature.

## Materials and methods

### Study design

This multicenter, observational, cross-sectional study enrolled consecutive patients from November 2017 to September 2025 at four movement disorders and cognitive-neurology centers. Four prespecified groups were studied: PD with PS (PD-PS), PD without PS (PD-noPS), DLB with PS (DLB-PS), and DLB without PS (DLB-noPS). No individual matching was imposed during recruitment because the aim was to preserve the natural clinical spectrum of both disorders; potential between-group imbalance was handled analytically. All participating centers used a shared operations manual, harmonized case-report forms, and joint rater training before recruitment started. The study protocol was approved by the ethics committee of each participating hospital. Written informed consent was obtained from all participants or, when decisional capacity was limited, from legally authorized representatives in accordance with the Declaration of Helsinki.

### Participants and diagnostic definitions

PD patients were diagnosed according to the Movement Disorder Society clinical diagnostic criteria (Postuma et al., 2015). DLB was diagnosed according to the fourth consensus report of the DLB Consortium (McKeith et al., 2017). To minimize diagnostic overlap across Lewy body disorders, the 1-year rule was applied throughout case adjudication: patients who fulfilled criteria for Parkinson’s disease dementia (PDD) were not included in the PD cohort. Participants were eligible if they were able to complete a standardized neurological examination, tolerate upright postural assessment, and provide reliable information either directly or through a knowledgeable caregiver. Exclusion criteria were as follows: (1) atypical or secondary parkinsonism; (2) major structural spinal disease likely to explain lateral trunk flexion, including scoliosis >10°, major vertebral fracture, or prior spinal fusion surgery; assessment was based on clinical history, physical examination, and, when indicated, review of available spinal imaging (standing X-ray or CT); when a structural abnormality was suspected and recent imaging was unavailable, standing whole-spine X-rays were obtained to measure the Cobb angle; exclusion was applied when the lateral trunk flexion was judged to be primarily attributable to a structural spinal condition; (3) primary dystonia, severe camptocormia (anterior trunk flexion ≥45°), antecollis (forward neck flexion ≥45°), inflammatory or inherited myopathy, ankylosing spondylitis, or other major musculoskeletal conditions affecting coronal posture. We excluded severe camptocormia and antecollis because these sagittal-plane deformities can independently alter gait biomechanics and postural control, potentially confounding the specific association between lateral trunk flexion. Moreover, reliable measurement of the lateral flexion angle becomes technically challenging when forward trunk flexion exceeds 45°. Patients with milder camptocormia or antecollis (<45°) without substantial interference with PS measurement were not excluded, but none were present in the final sample; (4) disabling stroke, defined as persistent hemiparesis (limb MRC grade ≤3), severe ataxia, major visual field deficit, or cortical sensorimotor loss that independently impaired gait or postural assessment, based on medical records and neurological examination; severe peripheral neuropathy, vestibular disease, or lower-limb orthopedic disorders precluding valid gait assessment; no enrolled patient had a history of strategic basal ganglia stroke; (5) acute psychosis unrelated to Lewy body disease, or severe medical instability at the time of assessment; and (6) recent initiation or substantial dose escalation, within the preceding 3 months, of medications known to induce dystonia or major postural instability. Stable use of clinically necessary antiparkinsonian drugs, cholinesterase inhibitors, memantine, antidepressants, and atypical antipsychotics was permitted and recorded in detail.

### Definition and standardized measurement of PS

PS was defined as at least 10° of lateral trunk flexion in the standing position (Doherty et al., 2013; Barone et al., 2016). The degree of lateral flexion was measured using a wall-mounted goniometer and verified from standardized digital photographs obtained in the coronal plane. Two trained raters independently reviewed all images; if the absolute difference between raters exceeded 3°, the case was adjudicated jointly. In addition to PS status, the following postural variables were recorded: angle of lateral flexion, side of flexion, and presence of back pain (Doherty et al., 2013).

### Demographic, clinical, and medication variables

For each participant, we recorded age at assessment, sex, years of education, age at symptom onset, disease duration, smoking and alcohol history, major vascular comorbidities, family history of PD or dementia, and the identity of the primary caregiver. Medication exposure was documented comprehensively, including levodopa preparations, dopamine agonists, amantadine, cholinesterase inhibitors, antidepressants, benzodiazepines, and antipsychotics. For antiparkinsonian treatment, the levodopa equivalent daily dose (LEDD) was calculated using established conversion factors (Tomlinson et al., 2010). To reduce short-term pharmacological noise, only participants on a stable medication regimen for at least 4 weeks were included.

### Cognitive, neuropsychiatric, and caregiver assessments

Cognitive assessment was performed face-to-face by neurologists trained to a common protocol. Global cognition was assessed with the Montreal Cognitive Assessment (MoCA) (Nasreddine et al., 2005). Visuoconstructive and executive performance was further assessed with the Clock Drawing Test (CDT) (Sunderland et al., 1989), and overall dementia severity was rated with the Clinical Dementia Rating (CDR) scale (Morris, 1993). Neuropsychiatric symptoms were assessed with the Neuropsychiatric Inventory (NPI) using caregiver-based interviews (Cummings et al., 1994). Caregiver burden was evaluated with the Zarit Burden Interview (ZBI) (Zarit et al., 1980). To improve multicenter consistency, interviewers used a standardized symptom-anchoring guide and entered item-level data.

### Motor severity, daily function, gait and falls

Motor impairment was quantified with part III of the Movement Disorder Society-sponsored revision of the Unified Parkinson’s Disease Rating Scale (MDS-UPDRS-III) (Goetz et al., 2008), and disease stage was rated with the Hoehn and Yahr (H&Y) scale (Hoehn and Yahr, 1967). Functional status was assessed using an activities of daily living (ADL) instrument, with higher scores indicating greater functional dependence (Roberts et al., 2017). The same motor and functional instruments were applied across PD and DLB whenever feasible in order to maximize comparability. Instrumented gait analysis was performed in all participants who could walk at least 8 m. Gait was assessed with a pressure-sensitive walkway system using a standardized protocol (Menz et al., 2004; Su et al., 2025c). Participants walked at their usual comfortable pace over the walkway for three trials, and the mean value was used for analysis. The gait variables were gait speed, stride length, gait symmetry index, and swing time. Falls were defined as unexpected events in which the participant came to rest on the ground, floor, or a lower level (Lamb et al., 2005). Fall history during the preceding 12 months was obtained from both patient and caregiver whenever possible. For the primary analyses, participants were classified as non-fallers or fallers; secondary analyses used a three-level categorization of no falls, occasional falls (1–2), and recurrent falls (3 or more). If a center maintained prospective fall calendars or follow-up diaries, those records were used to verify retrospective reporting.

### Statistical analysis

All statistical analyses and data management were performed using SPSS 26.0 for Mac (IBM Corporation, NY, USA). Continuous variables were expressed as mean ± standard deviation (SD) when normally distributed or as median (interquartile range) for non-normally distributed data. Between-group comparisons were performed using Student’s *t*-test for parametric data and the Mann–Whitney *U* test for nonparametric data. Categorical variables were summarized as frequencies (*n*) with percentages and analyzed using the *χ*^2^ test. The comparative analyses used regression models that included disease (PD vs. DLB), PS status (yes vs. no), and a disease-by-PS interaction term. Continuous outcomes were analyzed with multivariable linear models; binary outcomes were analyzed with logistic or ordinal logistic models. Model 2 additionally adjusted for MDS-UPDRS-III score and LEDD to address motor severity and medication burden. For neuropsychiatric outcomes, the sensitivity model additionally included exposure to cholinesterase inhibitors and antipsychotics. Because baseline global cognition differs between PD and DLB, secondary analyses either adjusted for CDR global score or standardized selected cognitive outcomes within disease strata to test whether observed interaction effects persisted beyond global dementia severity. To identify disease-specific predictors of PS, separate multivariable logistic regression models were built within the PD and DLB strata. Candidate predictors were selected on clinical grounds and from univariable screening at *p* < 0.10. If model instability was detected because of sparse events, penalized logistic regression was prespecified. Exploratory mediation analyses were planned when the required model assumptions were met. Because MDS-UPDRS-III includes item 3.13, which directly captures lateral trunk flexion, we performed a *post hoc* sensitivity analysis for the PD-specific logistic regression model by recalculating the UPDRS-III score after removing this item. All tests were two-sided, and *p* < 0.05 was considered statistically significant.

## Results

A total of 171 participants were included in the four-group comparison, comprising 50 patients with PD without PS (PD-nPS), 41 with PD and PS (PD-PS), 45 with DLB without PS (DLB-nPS), and 35 with DLB and PS (DLB-PS). The four groups were broadly comparable with respect to age, sex distribution, and educational attainment (Table 1). Within both PD and DLB, the presence of PS was accompanied by longer disease duration, poorer activities of daily living, and a markedly higher frequency of recurrent falls. The motor burden associated with PS was most pronounced in PD, with the PD-PS group showing the highest MDS-UPDRS-III scores and a shift toward more advanced H&Y stages. In contrast, the DLB-PS group carried the greatest cognitive and neuropsychiatric burden, with the lowest MoCA total score and the highest NPI total score among the four groups.

**Table 1:** Demographic and clinical characteristics of the four groups.

Variable	PD-nPS (*n* = 50)	PD-PS (*n* = 41)	DLB-nPS (*n* = 45)	DLB-PS (*n* = 35)	*p* value
Age, years	68.2 ± 7.5	69.1 ± 6.9	70.5 ± 7.1	71.2 ± 6.5	0.21
Male, *n* (%)	32 (64.0)	27 (65.9)	29 (64.4)	23 (65.7)	0.99
Education, years	11.2 ± 3.4	10.8 ± 3.8	10.5 ± 4.0	9.9 ± 3.5	0.34
Disease duration, years	5.4 ± 2.8	7.9 ± 3.2^***^	3.8 ± 2.0	5.6 ± 2.4^***††^	<0.001
Parkinsonism duration, years	5.4 ± 2.8	7.9 ± 3.2^***^	2.9 ± 1.8^††^	4.1 ± 2.0^*††‡^	<0.001
Lateral flexion angle, degrees	NA	14.5° ± 3.2^°^	NA	15.2° ± 3.8^°^	-
MDS-UPDRS-III	32.4 ± 10.5	47.8 ± 12.1^***^	28.5 ± 11.2	34.7 ± 10.9^†^	<0.001
H and Y stage, *n* (%)					<0.001
1.0	6 (12.0)	1 (2.4)	8 (17.8)	2 (5.7)	
1.5	8 (16.0)	2 (4.9)	10 (22.2)	3 (8.6)	
2.0	18 (36.0)	6 (14.6)	13 (28.9)	6 (17.1)	
2.5	12 (24.0)	8 (19.5)	8 (17.8)	8 (22.9)	
3.0	5 (10.0)	14 (34.1)	4 (8.9)	10 (28.6)	
4.0	1 (2.0)	8 (19.5)	2 (4.4)	5 (14.3)	
5.0	0 (0)	2 (4.9)	0 (0)	1 (2.9)	
ADL score	16.5 ± 5.2	24.8 ± 6.5^***^	18.2 ± 6.1	26.5 ± 7.2^***†^	<0.001
MoCA total score	24.2 ± 3.2	21.5 ± 3.8^***^	18.5 ± 4.5^††^	14.2 ± 4.1^***††‡^	<0.001
NPI total score	6.5 ± 5.1	10.2 ± 6.8^*^	12.8 ± 7.5^††^	22.5 ± 9.2^***††‡^	<0.001
Recurrent falls, *n* (%)	8 (16.0)	21 (51.2)^***^	10 (22.2)	18 (51.4)^***^	<0.001
LEDD, mg/day	485 ± 210	620 ± 245^*^	120 ± 95^††^	185 ± 110^††^	<0.001
Cholinesterase inhibitor use, *n* (%)	4 (8.0)	5 (12.2)	28 (62.2)^††^	26 (74.3)^***††^	<0.001

PD, Parkinson’s disease; PS, Pisa syndrome; DLB, dementia with Lewy bodies; MDS-UPDRS-III, Movement Disorder Society-Unified Parkinson’s Disease Rating Scale Part III; H&Y, Hoehn and Yahr; ADL, activities of daily living; MoCA, Montreal Cognitive Assessment; NPI, Neuropsychiatric Inventory; LEDD, levodopa equivalent daily dose. Lateral flexion angle is shown descriptively only. For non-PS groups, the angle was <10° by definition, but individual values were not quantitatively recorded; therefore, “NA” (not applicable) is reported; no inferential comparison was performed for this row. ^*^p < 0.05, ^**^p < 0.01, ^***^p < 0.001 compared to the corresponding non-PS group within the same disease; ^†^*p* < 0.05, ^††^*p* < 0.001 compared to PD-PS; ^‡^*p* < 0.001 compared to DLB-nPS.

Compared with the PD groups, both DLB groups had a shorter duration of parkinsonism and substantially lower levodopa equivalent daily dose, whereas cholinesterase inhibitor use was concentrated almost entirely in the DLB cohorts. Notably, the DLB-PS group combined postural deformity with advanced global cognitive impairment, heavy neuropsychiatric symptomatology, and frequent falls. Lateral flexion angle is presented descriptively because it forms part of the clinical definition of PS and is therefore not suitable for inferential group testing.

Adjusted two-way models showed that the clinical expression of PS was not uniform across Lewy body disorders (Table 2). All multivariable models were adjusted for disease duration, among other covariates, ensuring that the observed associations between PS and clinical outcomes are not attributable to longer disease duration in the PS groups. Beyond significant main effects of disease category and PS status, robust disease × PS interactions were observed for attention (*F* = 9.74, *p* = 0.002) and visuospatial/executive performance (*F* = 11.28, *p* < 0.001), indicating that the cognitive penalty associated with PS was greater in DLB than in PD. A similar interaction pattern was seen for hallucination severity (*F* = 8.56, *p* = 0.004), delusion severity (*F* = 5.83, *p* = 0.017), total neuropsychiatric burden (*F* = 6.45, *p* = 0.012), and caregiver burden (*F* = 7.82, *p* = 0.006).

**Table 2:** Adjusted two-way models examining the main effects of disease category, PS status, and their interaction.

Outcome	Disease effect (PD vs. DLB)	PS effect (no PS vs. PS)	Disease × PS interaction
Attention score (MoCA subscore)	*F* = 8.22, *p* = 0.005	*F* = 15.36, *p* < 0.001	*F* = 9.74, *p* = 0.002
Visuospatial/executive score (CDT)	*F* = 12.05, *p* < 0.001	*F* = 18.44, p < 0.001	*F* = 11.28, *p* < 0.001
Hallucination severity (NPI item)	*F* = 10.33, *p* = 0.002	*F* = 13.87, *p* < 0.001	*F* = 8.56, *p* = 0.004
Delusion severity (NPI item)	*F* = 6.21, *p* = 0.014	*F* = 9.45, *p* = 0.002	*F* = 5.83, *p* = 0.017
NPI total score	*F* = 15.67, *p* < 0.001	*F* = 20.11, *p* < 0.001	*F* = 6.45, *p* = 0.012
Caregiver burden (ZBI)	*F* = 12.89, *p* < 0.001	*F* = 22.34, *p* < 0.001	*F* = 7.82, *p* = 0.006
MDS-UPDRS-III total score	*F* = 9.56, *p* = 0.002	*F* = 28.45, *p* < 0.001	*F* = 5.21, *p* = 0.024
Gait speed (m/s)	*F* = 5.44, *p* = 0.021	*F* = 18.77, *p* < 0.001	*F* = 6.92, *p* = 0.009
Stride length (m)	*F* = 3.98, *p* = 0.048	*F* = 22.13, *p* < 0.001	*F* = 7.34, *p* = 0.008
Gait symmetry (ratio)	*F* = 4.12, *p* = 0.044	*F* = 15.89, *p* < 0.001	F = 6.78, *p* = 0.010
Recurrent falls	OR = 0.85 [0.48–1.52], *p* = 0.59	OR = 4.45 [2.21–8.96], *p* < 0.001	OR = 2.98 [1.32–6.73], *p* = 0.009

PD, Parkinson’s disease; DLB, dementia with Lewy bodies; PS, Pisa syndrome; MoCA, Montreal Cognitive Assessment; CDT, Clock Drawing Test; NPI, Neuropsychiatric Inventory; ZBI, Zarit Burden Interview; MDS-UPDRS-III, Movement Disorder Society-Unified Parkinson’s Disease Rating Scale Part III. Continuous outcomes were analyzed using adjusted general linear models; recurrent falls was analyzed using multivariable logistic regression. All models were adjusted for age, sex, education, disease duration, and levodopa equivalent daily dose.

**Figure 1:**
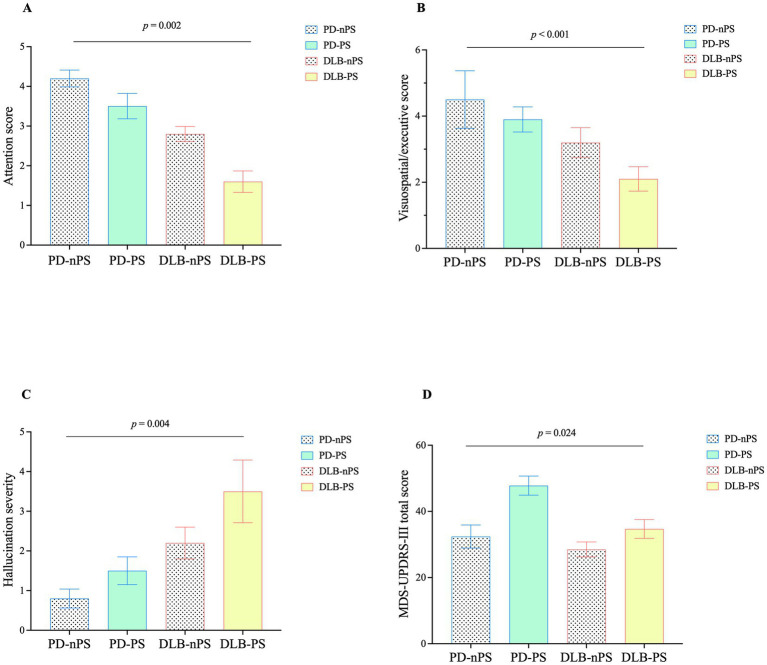
Disease-by-Pisa syndrome interaction for selected outcomes. Bars represent mean ± SD. Four groups: PD without PS (PD-nPS, *n* = 50), PD with PS (PD-PS, *n* = 41), DLB without PS (DLB-nPS, *n* = 45), DLB with PS (DLB-PS, *n* = 35). **(A)** Attention subscore; **(B)** Visuospatial/executive score; **(C)** Hallucination severity; **(D)** MDS-UPDRS-III total score. *p* values for disease × PS interaction (from two-way models adjusted for age, sex, education, disease duration, and LEDD): attention *p* = 0.002, visuospatial/executive *p* < 0.001, hallucination *p* = 0.004, MDS-UPDRS-III *p* = 0.024.

The interaction terms for motor and gait outcomes pointed in a different direction. Significant disease × PS interactions were also present for MDS-UPDRS-III (*F* = 5.21, *p* = 0.024), gait speed (*F* = 6.92, *p* = 0.009), stride length (*F* = 7.34, *p* = 0.008), and gait symmetry (*F* = 6.78, *p* = 0.010). For recurrent falls, PS remained the dominant main effect (OR = 4.45, 95% CI 2.21–8.96; *p* < 0.001), but the disease × PS interaction was likewise significant (OR = 2.98, 95% CI 1.32–6.73; p = 0.009). The key interaction patterns for attention, visuospatial/executive function, hallucinations, and motor severity are visualized in Figure 1.

**Figure 2:**
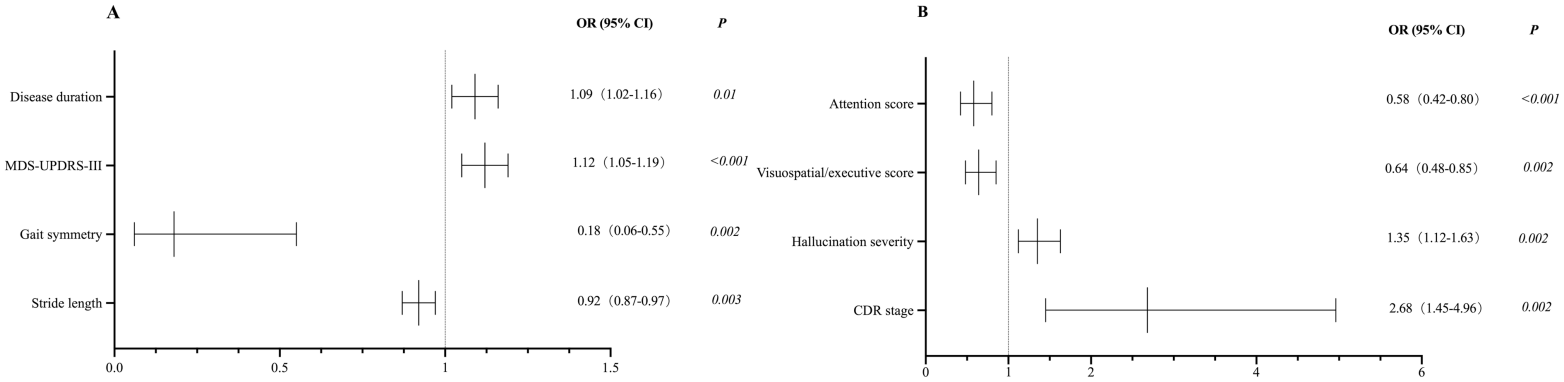
Disease-specific independent predictors of Pisa syndrome. Forest plots show odds ratios (OR) and 95% confidence intervals from multivariable logistic regression models. **(A)** Shows predictors for PD (disease duration, MDS-UPDRS-III, gait symmetry, stride length), and the **(B)** shows predictors for DLB (attention score, visuospatial/executive score, hallucination severity, CDR stage). All models adjusted for age, sex, education, disease duration, and LEDD.

When the two diseases were modeled separately, different predictors of PS emerged (Table 3). In PD, the presence of PS was independently associated with longer disease duration (OR = 1.09, 95% CI 1.02–1.16; *p* = 0.01), higher MDS-UPDRS-III scores (OR = 1.12, 95% CI 1.05–1.19; *p* < 0.001), lower gait symmetry (OR = 0.18, 95% CI 0.06–0.55; *p* = 0.002), and shorter stride length (OR = 0.92, 95% CI 0.87–0.97; *p* = 0.003). A sensitivity analysis that excluded the posture item (item 3.13) from the UPDRS-III score yielded a slightly attenuated but still significant association (OR = 1.08, 95% CI 1.03–1.14; *p* = 0.002). The DLB model showed a distinctly different pattern. Lower attention scores (OR = 0.58, 95% CI 0.42–0.80; *p* < 0.001), lower visuospatial/executive scores (OR = 0.64, 95% CI 0.48–0.85; p = 0.002), greater hallucination severity (OR = 1.35, 95% CI 1.12–1.63; *p* = 0.002), and a higher CDR stage (OR = 2.68, 95% CI 1.45–4.96; *p* = 0.002) were independently associated with PS, whereas disease duration and MDS-UPDRS-III were not significant. Although statistically significant, the odds ratios for individual predictors were modest in magnitude. Figure 2 presents forest plots of these disease-specific independent predictors for PD (A panel) and DLB (B panel).

**Table 3:** Disease-specific multivariable logistic regression models for the presence of PS.

Predictor	PD model OR (95% CI)	*p* value	DLB model OR (95% CI)	*p* value
Disease duration	1.09 (1.02–1.16)	0.01	1.08 (0.96–1.21)	0.19
MDS-UPDRS-III (full scale)	1.12 (1.05–1.19)	<0.001	1.07 (0.96–1.19)	0.21
MDS-UPDRS-III (excluding item 3.13)^a^	1.08 (1.03–1.14)	0.002	–	–
Attention score	NR	–	0.58 (0.42–0.80)	<0.001
Visuospatial/executive score	NR	–	0.64 (0.48–0.85)	0.002
Hallucination severity	NR	–	1.35 (1.12–1.63)	0.002
Gait symmetry	0.18 (0.06–0.55)	0.002	NR	–
Stride length	0.92 (0.87–0.97)	0.003	NR	–
CDR stage^b^	NA	–	2.68 (1.45–4.96)	0.002

PD, Parkinson’s disease; DLB, dementia with Lewy bodies; MDS-UPDRS-III, Movement Disorder Society-Unified Parkinson’s Disease Rating Scale Part III. CDR, Clinical Dementia Rating; NA, not applicable; NR, not retained. Each disease-specific model was additionally adjusted for age, sex, education, and levodopa equivalent daily dose. Continuous predictors were entered on their original measurement scales.

a Because MDS-UPDRS-III includes item 3.13 (Posture), the PD model was re-run after removing this item.

b CDR was modeled as an ordinal variable (0.5, 1, 2, 3); the reported OR represents the change in odds of PS for each one-level increase in CDR stage.

Mediation analyses were shown in Table 4. In DLB, the association between PS and recurrent falls was partly transmitted through an attention/visuospatial composite. The total effect of PS on recurrent falls was significant (*β* = 0.66, *p* < 0.001), and the indirect effect through the cognitive mediator remained robust (*β* = 0.24, 95% CI 0.12–0.39), accounting for 36.4% of the total effect. The direct path from PS to recurrent falls remained significant after inclusion of the mediator (*β* = 0.42, *p* < 0.05), indicating partial rather than complete mediation. In a second DLB-oriented model, hallucination burden partially mediated the association between PS and caregiver burden, with an indirect effect of *β* = 0.31 (95% CI 0.16–0.48), representing 41.9% of the total effect. In PD, by contrast, gait asymmetry and stride impairment accounted for a larger share of the link between PS and recurrent falls. The indirect effect was *β* = 0.42 (95% CI 0.23–0.63), corresponding to 60.9% of the total effect. The gait mediator was entered, and the direct association between PS and recurrent falls was attenuated and no longer statistically significant (*β* = 0.27).

**Table 4:** Mediation models linking PS to falls and caregiver burden.

Model	Total effect (c)	Direct effect (c’)	Indirect effect (ab)	95% bootstrap CI	Proportion mediated
DLB: PS → attention/visuospatial composite → recurrent falls	*β* = 0.66^**^	*β* = 0.42^*^	*β* = 0.24	0.12–0.39	36.4%
DLB: PS → hallucination burden → caregiver burden	*β* = 0.74^**^	*β* = 0.43^*^	*β* = 0.31	0.16–0.48	41.9%
PD: PS → gait asymmetry / stride impairment → recurrent falls	*β* = 0.69^**^	*β* = 0.27	*β* = 0.42	0.23–0.63	60.9%

PD, Parkinson’s disease; DLB, dementia with Lewy bodies; PS, Pisa syndrome. *β* denotes the regression coefficient from the mediation model. For models with recurrent falls as the outcome, coefficients are presented on the fitted model scale and should not be interpreted as odds ratios. Bootstrap confidence intervals were estimated with 5,000 resamples. All models were adjusted for age, disease duration, and levodopa equivalent daily dose. ^*^p < 0.05, ^**^p < 0.001.

## Discussion

The main message of this study is that PS should not be treated as a uniform axial complication across Lewy body disorders. Under a harmonized assessment framework, although PS in both PD and DLB marked a more disabled subgroup—with longer disease duration, worse daily function, and substantially more recurrent falls—the pattern of coexisting deficits diverged in a systematic way. In the adjusted interaction models, DLB-PS was characterized by a disproportionate penalty in attention, visuospatial/executive performance, psychosis-related symptoms, and caregiver burden, whereas PD-PS was more strongly anchored to motor severity and gait disorganization. Recent evidence has already shown that axial postural abnormalities in PD are common, clinically meaningful, and phenotypically heterogeneous rather than reducible to a single mechanism (Barone et al., 2016; Tinazzi et al., 2016; Cao et al., 2023). Quantitative multicenter work has linked axial postural deviation with gait disorganization in PD (Pongmala et al., 2022). Our findings extend that idea across the Lewy body spectrum and suggest that PS is best interpreted as a disease-contextual sign: its outward appearance may be similar, but its clinical meaning depends on the network vulnerability in which it emerges.

The strongest interaction effects involved attention, visuospatial or executive performance, hallucination severity, total neuropsychiatric burden, and caregiver burden, and within DLB the independent correlates of PS were cognitive and psychosis-related rather than motor. This pattern argues against viewing DLB-PS as a simple consequence of axial rigidity or late-stage frailty. The more plausible interpretation is that PS in DLB sits downstream of posterior cortical and attentional network failure, where impaired visual sampling, unstable internal models of body position, and reduced capacity to reweight sensory cues may amplify lateral trunk deviation during stance and gait (Adele et al., 2024; Su et al., 2025a). A 2024 imaging study linked gait dysfunction in DLB to specific neural substrates (Sainsily-Cesarus et al., 2024); current research emphasizes that visual dysfunction in DLB extends well beyond acuity, involving dorsal-stream, occipital, and higher-order visuoperceptual disturbances (Devenyi and Hamedani, 2024). Longitudinal data further suggest that visual hallucinations in DLB are foreshadowed by attention and visuospatial deficits (Pezzoli et al., 2021). Read together with our results, these studies make it reasonable to think of DLB-PS as the visible motor footprint of a broader cognition-neuropsychiatry-amplified syndrome, rather than as a posture problem in isolation.

The clinical weight of this DLB-oriented phenotype is underscored by the caregiver findings. In our two-way models, caregiver burden was not merely a parallel consequence of dementia severity; it tracked the neuropsychiatric dimension of PS, and hallucination burden partly mediated the association between PS and caregiver burden. This is similar to recent DLB study showing that neuropsychiatric symptoms are among the strongest drivers of caregiver strain, including in longitudinal settings where the persistence or emergence of anxiety, apathy, or psychosis can worsen burden over time (Hayashi et al., 2025; Su et al., 2025a). The broader burden research in DLB points in the same direction, identifying behavioral and psychological symptoms as key determinants of family stress and care disruption (Toledo et al., 2023). Clinically, this means that the appearance of PS in DLB should prompt more than a musculoskeletal examination. It should trigger structured screening for hallucinations, fluctuations, attentional instability, fall exposure, and caregiver overload because the postural sign may be signaling a broader phase of disease escalation.

By contrast, the PD findings point to a different emphasis. In PD, PS was independently associated with disease duration, motor severity, gait symmetry, and stride length, and the effect of PS on recurrent falls was largely mediated by gait asymmetry and stride impairment, while cognitive and psychosis-related variables did not remain in the disease-specific model. That pattern is more consistent with a sensorimotor disorder of axial alignment: asymmetric basal ganglia output, impaired integration of vestibular and proprioceptive cues, abnormal verticality perception, and progressive musculoskeletal adaptation (Castrioto et al., 2014; Barone et al., 2016; Tinazzi et al., 2016; Artusi C. A. et al., 2023). Quantitative studies have shown a tight coupling between gait abnormalities and axial postural deviations in PD (Pongmala et al., 2022). Imaging work has identified right posterior hypometabolism in PD patients with PS, supporting a contribution from disturbed body schema and spatial perception rather than pure dystonia or fixed skeletal change alone (Biassoni et al., 2023).

The association between gait dysfunction and higher-order cognitive control in PD is now well recognized, particularly for locomotor phenomena that depend on continuous adjustment (Monaghan et al., 2023). Our mediation analysis fits that framework: in PD, the pathway from PS to recurrent falls was carried largely through gait asymmetry and stride impairment, suggesting that the real clinical danger of PS lies not simply in how the patient stands but in how the postural distortion destabilizes dynamic locomotion. This profile is consistent with the established PD studies, which have emphasized axial motor deterioration, impaired sensory integration, altered verticality perception, and disturbed body schema as central components of PS pathophysiology (Castrioto et al., 2014; Tinazzi et al., 2015, 2016; Barone et al., 2016; Huh et al., 2018; Liu et al., 2019). Importantly, this does not mean that cognition is irrelevant in PD-PS. Previous studies have shown that PD with PS can also exhibit selective attentional and visuospatial abnormalities (Vitale et al., 2016; Artusi et al., 2019; Artusi C. et al., 2023). Our result suggests a difference in weighting rather than a difference in kind: cognition may still contribute in PD, but the dominant clinical expression is more clearly routed through gait and postural control.

Falls are common and consequential in Lewy body disorders, yet the mechanisms that connect lateral trunk flexion to fall risk are unlikely to be identical across diseases (Gonzalez et al., 2023; Adele et al., 2024; Su et al., 2025c). Our data support that view. In DLB, PS may increase fall vulnerability partly because the same patients are less able to allocate attention, monitor spatial relationships, and compensate for postural misalignment. In PD, the route appears more biomechanical and sensorimotor, with asymmetric gait and shortened stride providing a more direct bridge from axial deformity to repeated falls. This distinction matters because the same bedside sign—lateral trunk flexion—may be pointing clinicians toward different forms depending on the diagnostic context.

The uniform management pathway for PS risks missing the main source of vulnerability in each disease. In DLB, PS should probably be treated as a high-risk clinical marker for falls, neuropsychiatric destabilization, and caregiver strain; posture assessment may therefore need to be paired with targeted cognitive, visual, and behavioral screening. In PD, management is more likely to be effective when anchored in gait-focused rehabilitation, postural realignment strategies, medication review, and selective interventions directed at axial dysfunction. Therefore, treatment of axial postural abnormalities in parkinsonism is necessarily multimodal and that response heterogeneity is substantial, especially once deformity becomes chronic or structurally fixed (Gandolfi et al., 2024). From a translational standpoint, we argue for personalized rather than label-based management: DLB-PS calls for closer monitoring of cognition, hallucinations, falls, and caregiver needs, whereas PD-PS calls for earlier recognition of gait disorganization and earlier intervention before compensatory postural changes become entrenched.

PS should be used as a screening flag rather than recorded merely as a postural descriptor. In DLB, the appearance of PS should prompt a focused review of attention, visuospatial function, hallucinations, fluctuations, fall history, and caregiver strain, even when the motor examination has not changed dramatically (McKeith et al., 2017; Su et al., 2022; Adele et al., 2024; Su et al., 2025a,b,c). In PD, PS should trigger careful gait assessment, recurrent-fall surveillance, medication review, and early referral for posture- and balance-oriented rehabilitation (Barone et al., 2016; Tinazzi et al., 2016; Al-Wardat et al., 1996; Carlo Alberto et al., 2023). The DLB-PS phenotype may benefit most from combined cognitive-behavioral monitoring, caregiver education, environmental fall prevention, and judicious optimization of cognition- or psychosis-directed therapy, whereas the PD-PS phenotype may require greater emphasis on sensorimotor retraining, asymmetric gait correction, and axial motor management.

Establishing whether PS is a uniform or disease-contextual phenomenon across Lewy body disorders has direct clinical implications. If the clinical signature of PS differs between PD and DLB, then the same postural sign should trigger different diagnostic and management pathways depending on the underlying diagnosis. For PD-PS, clinicians would prioritize gait assessment, fall prevention, and sensorimotor rehabilitation; for DLB-PS, structured screening for attentional deficits, visual hallucinations, and caregiver burden would be warranted, even in the absence of marked motor worsening. Thus, the present comparison is not merely descriptive but aims to provide evidence-based guidance for phenotype-specific evaluation and treatment of a common axial deformity across the Lewy body spectrum.

Several limitations deserve acknowledgment. First, the cross-sectional design precludes causal inference; the observed pathways should be interpreted as clinically plausible models rather than proof of temporal sequence. Second, although the multicenter design improves generalizability, it introduces treatment heterogeneity, and retrospective fall ascertainment remains vulnerable to recall bias despite caregiver corroboration. Third, residual confounding by medication and disease stage remains possible despite analytical adjustment, and we did not have access to several mechanistic measures that would have sharpened interpretation, such as formal vestibular testing, body-verticality paradigms, advanced neuroimaging, or quantitative trunk electromyography. Fourth, we excluded patients with severe camptocormia or antecollis (≥45°) to avoid confounding from sagittal-plane axial deformities, which may alter gait and postural control through distinct biomechanical pathways. While this choice enhanced the internal validity and phenotypic homogeneity of our PS definition, it limits the generalisability of our findings to patients with mixed axial phenotypes. Future multicentre studies specifically designed to compare isolated PS versus PS with camptocormia/antecollis across Lewy body disorders are needed to determine whether our disease-specific signatures persist in more complex presentations. Fifth, the MDS-UPDRS-III includes item 3.13, which directly assesses lateral trunk flexion; therefore, the higher UPDRS-III scores in PD-PS compared with PD-noPS may be partially inflated by the postural deformity itself. However, our sensitivity analysis excluding this item showed that the association between the modified UPDRS-III score and PS remained statistically significant, albeit with a slightly reduced effect size, suggesting that broader motor severity beyond posture contributes to the observed difference. Finally, we excluded PDD to maintain diagnostic clarity. However, DLB and PD are phenotypes on the same Lewy body spectrum, sharing α-synuclein pathology. By excluding PDD, we did not address the full continuum. Thus, we cannot conclude that PS is unrelated to cognition in PD or that PS is unrelated to motor severity in DLB. DLB itself is heterogeneous and frequently accompanied by co-pathologies that can reshape cognition, hallucinations, gait, and axial control (Toledo et al., 2023). PS might show both cognitive and motor associations in PDD or DLB with pronounced parkinsonism. Our findings are applicable to “pure” phenotypic extremes; generalization to mixed phenotypes requires future studies. Therefore, longitudinal studies covering the full spectrum—from PD without dementia to PDD to DLB—are needed to determine whether PS marks an inflection point and whether the distinct PD/DLB pathways converge or switch as cognitive and motor impairment co-progress.

## Conclusion

PS across Lewy body disorders should not be viewed as a unitary postural complication. In the present cross-sectional study, DLB-PS showed stronger relative associations with cognitive and neuropsychiatric features, whereas PD-PS showed stronger relative associations with motor and gait impairment. However, the odds ratios for individual predictors were modest, suggesting that PS is a multifactorial clinical sign rather than a deterministic marker of any single domain. Recognizing these distinct but moderate clinical signatures may improve case finding, fall-risk stratification, caregiver counseling, and the tailoring of rehabilitation and pharmacological strategies. The modest effect sizes imply that PS should be interpreted in conjunction with other clinical features rather than in isolation. Longitudinal studies with multimodal phenotyping are needed to determine whether these disease-specific PS patterns predict differential disease trajectories or treatment responses.

## Data Availability

The original contributions presented in the study are included in the article/supplementary material, further inquiries can be directed to the corresponding authors.
